# Eruption Timing and Sequence of Primary Teeth in a Sample of Romanian Children

**DOI:** 10.3390/diagnostics12030606

**Published:** 2022-02-28

**Authors:** Emilia Ogodescu, Malina Popa, Claudia Isac, Raluca Pinosanu, Diana Olaru, Anca Cismas, Anca Tudor, Mariana Miron

**Affiliations:** 1 Pediatric Dentistry Research Center, Department of Pediatric Dentistry, Faculty of Dentistry “Victor Babes”, University of Medicine and Pharmacy, Eftimie Murgu Square No. 2, 300041 Timisoara, Romania; 2 Department of Dentistry, Emergency Municipal Clinical Hospital of Timisoara, Str. Hector No. 2A, 300041 Timisoara, Romania; ralu.pinosanu@gmail.com (R.P.); olaru_beatrice_92@yahoo.com (D.O.); cismasanca@gmail.com (A.C.); 3 Department of Functional Sciences, Faculty of Medicine “Victor Babes”, University of Medicine and Pharmacy, Eftimie Murgu Square No. 2, 300041 Timisoara, Romania; 4 Department of Oral Rehabilitation and Dental Emergencies, Faculty of Dentistry “Victor Babes”, University of Medicine and Pharmacy, Eftimie Murgu Square No. 2, 300041 Timisoara, Romania; miron.mariana@umft.ro

**Keywords:** eruption timing, eruption sequence, number of teeth, primary teeth, reference values, questionnaires, healthy children

## Abstract

Teething is considered a significant event in the growth and development of the child by parents and especially by paediatric dentists and forensic scientists. They constantly need an “up-to-date mirror” of its variability for different geographic regions. The aims of the current study were to determine the timing and sequence of primary tooth emergence, and to establish a correlation between tooth eruption and general growth and external parameters in a sample of Romanian children. This study included 70 healthy children (53% girls and 47% boys), and the analysed data resulted from the questionnaires completed by parents during the whole process. General and specific data on primary teeth eruption were obtained. The differences between sexes were insignificant, except for the maxillary and mandibular canines (*p* = 0.047 and *p* = 0.018) and lower temporary second molars (*p* < 0.001), which were significantly increased in boys. The differences found between the two hemiarches were insignificant (*p* = 0.197). The mean age of eruption of the first tooth erupted was 7.07 ± 1.990 months. The unpaired *t*-test was used for comparison of the timings of eruption of the first primary teeth, according to the number of previous births and type of diet, and it was insignificant (*p* = 0.665 and *p* = 0.983 respectively).

## 1. Introduction

Primary tooth eruption is a complex and highly regulated process in which teeth enter the mouth and become visible during a certain time period [1]. The formation and development of human primary teeth begins at the end of the fifth week of gestation. Teeth are formed in the upper and lower jaw through mutual, subtle and sophisticated interactions between the dental epithelium and oral ectomesenchyme involving the expression of several tooth-related genes [1,2,3]. Teething is considered a significant event in the growth and development of the child by parents and health care professionals, especially by paediatric dentists and forensic scientists [3,4,5,6]. Its importance extends in other fields, such as physical anthropology and demographic studies, and it is considered “a milestone” on the complex path of human growth [7,8,9,10]. Poureslami et al. (2015) determined that the eruption timing for the first primary tooth had a correlation with the first permanent tooth eruption timing [11]. Tooth emergence may have particular significance in relation to early childhood caries, because the likelihood of colonisation by mutans streptococci increases with the number of teeth present, contributing to high levels of caries in some populations [12,13].

The eruption timing of deciduous teeth is usually between 4 and 36 months. The typical order of tooth emergence is: central incisor, lateral incisor, first molar, canine, and second molar in the maxillary and mandibular arch [14,15]. Variations in the eruption timing of the first primary tooth (ETFPT) are considered multifactorial. Eruption is under genetic control, and the estimates of narrow-sense heritability are over 70% [1,16]. Furthermore, genome-wide association studies (GWAS) have identified some candidate genes associated with tooth development [1,17]. There are ethnicity and sexrelated differences in the timing of primary tooth eruption [1,6,12,13,18,19]. However, external environmental factors also make significant contributions to the timing of primary tooth eruption. Maternal exposure to tobacco during pregnancy [1,20,21,22], infant birth weight [1,23,24,25], birth length [1,26], nutritional state at birth and at postnatal time points [1,17], gestational age [1,18,23], method of infant feeding [1,21,27,28], and socioeconomic situation [1,29] have been reported to be significant determinants of primary teeth eruption. An estimation of the eruption schedule can be a very valuable asset for diagnosis and treatment planning during the developmental years, including for the diagnosis of developmental oral disturbances during the early childhood period [8,30,31,32,33]. Dental age is the key factor for the implementation of cavity-prevention programs such as topical fluoride application, age estimation in forensic sciences, and anthropology [9,10,31,34,35,36].

The last reference values for primary teeth eruption in our region were updated by E. Bratu in 1982. She considered that the paediatric dentist constantly needs an “up-to-date mirror” of the variability of tooth eruption for different geographic regions [37]. The aims of this study were: (1) to determine the most common timing and sequence of primary tooth emergence in a sample of Romanian children; (2) to compare the results between sexes, arches and hemiarches; (3) to determine the number of teeth at different ages; (4) to determine the correlation between the age of eruption of the first primary tooth/number of teeth at one year, and different general growth and external parameters; (5) to make a comparison between the eruption timings of the first primary teeth according to the child’s place in the sequence of births and type of diet, and (6) to compare current data with previous studies in different populations.

## 2. Materials and Methods

### 2.1. Instruments Used in the Study and Variables Recorded

**Figure A1:**
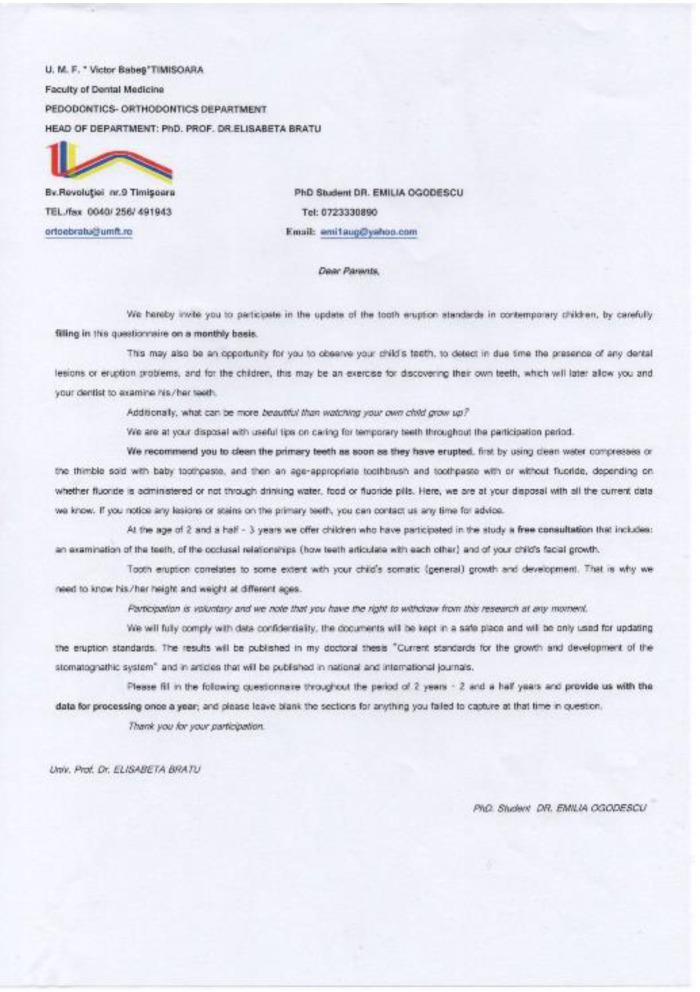
First page of the questionnaire (presentation of the study for the parents).

**Figure A2:**
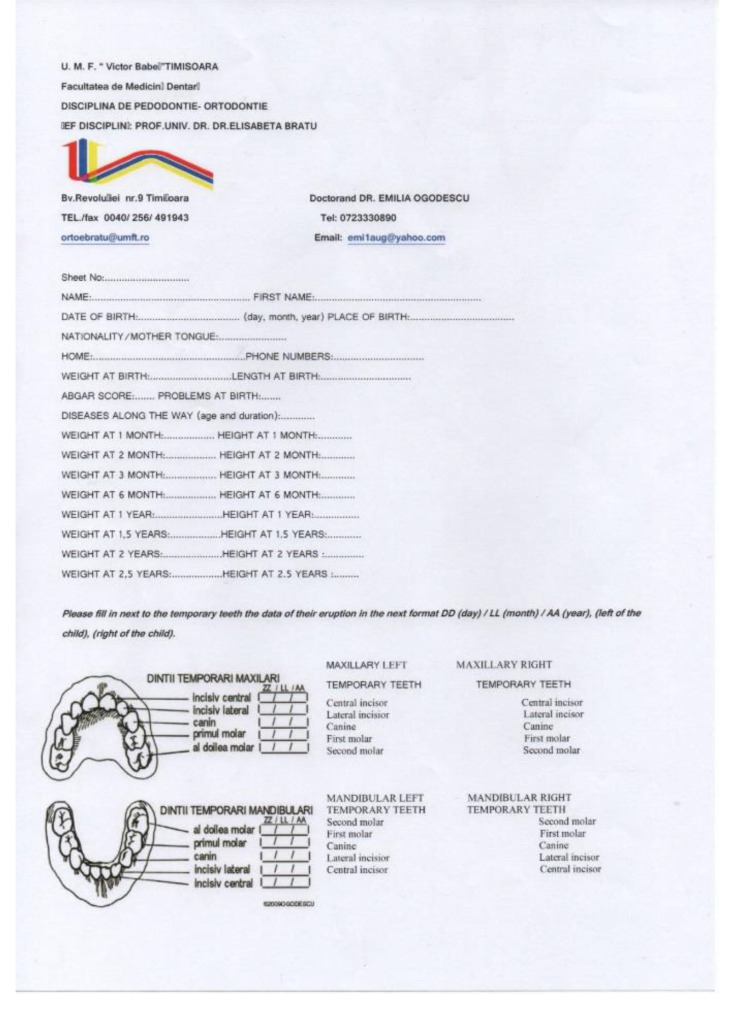
Second page of the questionnaire (used to collect the data for our study).

In the current study on the eruption of temporary teeth, 200 *questionnaires* were distributed to parents of children aged between a few days after birth and 1 year. The first page included general information about the study and the ethical guidelines, information about the oral hygiene measures for the primary teeth and fluoride prophylaxis (Figure A1). The second page of the questionnaire included the general data of the child: name, surname, date of birth (day, month, year), place of birth, nationality of the child, place of living and telephone number. This was followed by data on the general health of the child and certain diseases that may occur during the recording of the data. To these, information was added about the first anthropometric data (weight and length) at birth, Apgar score (Appearance, Pulse, Grimace, Activity, and Respiration), possible problems occurring at birth, age of the mother at birth, number of previous births, and data about the child’s nutrition (natural, artificial, mixed), as well as the number of months for which the child was breast-fed or had a mixed diet (Figure A2).

### 2.2. Parents Instruction and Procedure Calibration

The parents performed the first examination after they were trained and calibrated. We met every single parent for training before they started completing the questionnaires, and we communicated with parents through the study by phone, email, and through the intraoral pictures they sent. Observation of the child’s oral cavity was to be made weekly, and the recording was to be made at the time when the change was noticed. This was accompanied by the palpation of alveolar ridges, bucally and lingually.

Parents benefited from a helpful scheme of temporary teeth. Each tooth was considered erupted when it pierced the oral mucosa with any of its parts (incisal edge or a cuspid) and became visible in the oral cavity. Capturing the exact moment of the dental eruption was one of the most important rules to be observed when completing this questionnaire. It was considered ideal for the recording to be made in the week when the tooth erupted. For this, it was recommended for the examination to be performed in good lighting conditions and by playing, with a lot of patience.

### 2.3. Study Protocol

The questionnaires were distributed to parents from different social groups with the help of other colleagues (dentists, general medical practitioners, and paediatricians). The inclusion criteria were: healthy children of Romanian nationality. The exclusion criteria were congenital syndromes and other known general pathologies.

Participation in the study was voluntary. Written informed consent was obtained from all of the participants’ parents. The study was carried out in accordance with the Declaration of Helsinki and it was approved by the Institutional Ethics Committee (No 04/15.09.2008).

The first data were collected after a period of one year, and a further collection was conducted at an interval of three months. For accuracy control, every child was invited to participate in a cross-sectional study involving one dental check-up during the study, according to both parents’ decision. Ultimately, only 36 children were evaluated once in the dental practice in the parent’s presence, from which 17 girls (47%) and 19 boys (53%), with the age range between 1 and 3.4 years, having the mean age of 1.52 ± 0.6. Each temporary tooth present received a score: the value 0—the moment of dental eruption (the tooth penetrates the gum with a portion or with the entire incisal edge when we refer to the incisors, or with one or two cusps for the rest of the teeth); the value 0.25—if a quarter of the height of the tooth was visible; the value 0.5—if about half of the height of the tooth was visible; or the value 1—if more than half was erupted to the entire clinical crown. Absent teeth did not receive any score.

### 2.4. Sample Description

From the distributed questionnaires, only 70 were received back. According to this, our sample included a number of 70 children, of which 37 were girls, representing 53%, and 33 boys, representing 47%. For the sample size we made a G Power test belonging to the *t*-tests family (difference between two independent groups) with two tails, 80% power, a 0.05 significance level, and 0.7 effect size.

### 2.5. Data Analysis

The data were recorded in an examination sheet and subsequently transferred to the Excel database, which included: the name, age and sex of the child, registration code, date of birth, date of registration, age (calculated by Excel in years with one decimal place), height and weight at the date of registration, code given to each temporary tooth, and number of temporary teeth present for each registered child (automatically calculated using the calculation formula applied in the program). The teeth numbering system utilised in this study was the internationally recognised two-digit FDI World Dental Federation (Fédération Dentaire Internationale) system.

All the recorded data were processed within the Discipline of Biostatistics and Medical Informatics of “Victor Babes” University of Medicine and Pharmacy in Timisoara with the help of the SPSS v17 program. Descriptive statistics were calculated for the subjects’ characteristics, as well as the age of eruption of each primary tooth (mean and standard deviation). Because all numerical data were normally distributed (Shapiro–Wilk Test of Normality, *p* > 0.05), we used the unpaired *t*-test for comparing the independent values between sexes, between type of nutrition or number of preceding births, and the linear correlation analysis using the Pearson correlation coefficient in order to determine the correlation between the eruption timing of the first primary tooth or the number of teeth erupted at one year and the general growth parameters.

## 3. Results

After the statistical processing of the data resulting from the current study, the following results were obtained, which can be grouped into two main categories as follows:

- General data related to the birth, general development, and feeding of children from the examined group: distribution of children in the group by year of birth, percentage of children born from primiparous and multiparous mothers, Apgar score, average age of the children’s mothers at birth, and distribution of children by the mother’s age at birth, height, and weight at birth and at different moments; food-related data: natural, artificial or mixed diet;
- Specific data for primary teeth eruption: mean age of eruption for each tooth and for pairs of homologous primary teeth for both sexes, mean sequence of eruption, comparison of mean ages of eruption between sexes and between the two jaws, and, on the same jaw, between the two hemiarches; average number of teeth present at different ages; average age of eruption of the first primary tooth and different correlations between this mean age and the general parameters.

### 3.1. General Data for the Current Study Sample

**Figure 1:**
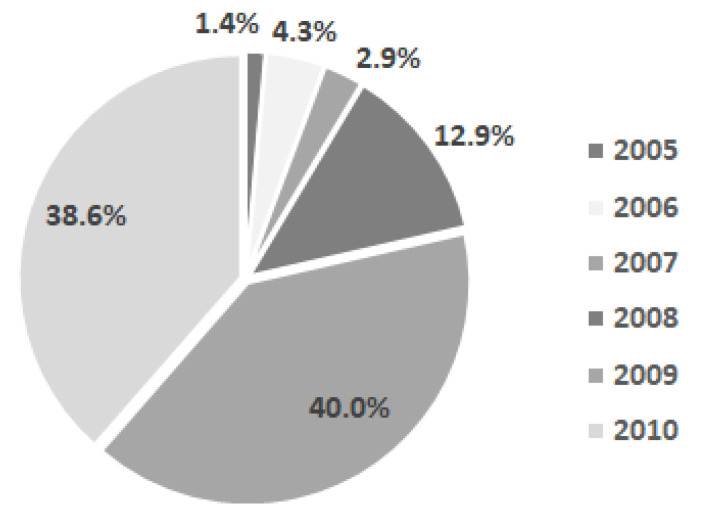
Distribution of our sample according to the year of their birth.

Most of the children in the study group were born in 2009 and 2010, nine children were born in 2008, and very few between 2005 and 2007 (Figure 1).

**Figure 2:**
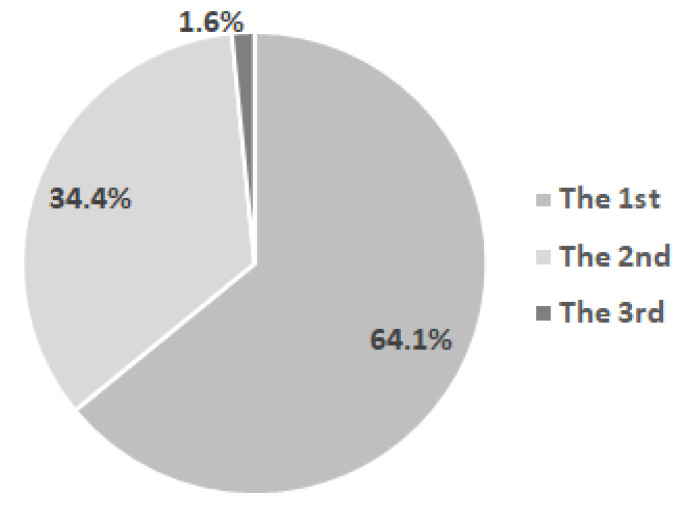
Distribution of children by place in the sequence of sibling births.

The distribution of children by place in the sequence of sibling births indicates that two-thirds of the children included in the study were the first-born, and about one-third of the children were the second-born, with one child being the third-born (Figure 2).

**Figure 3:**
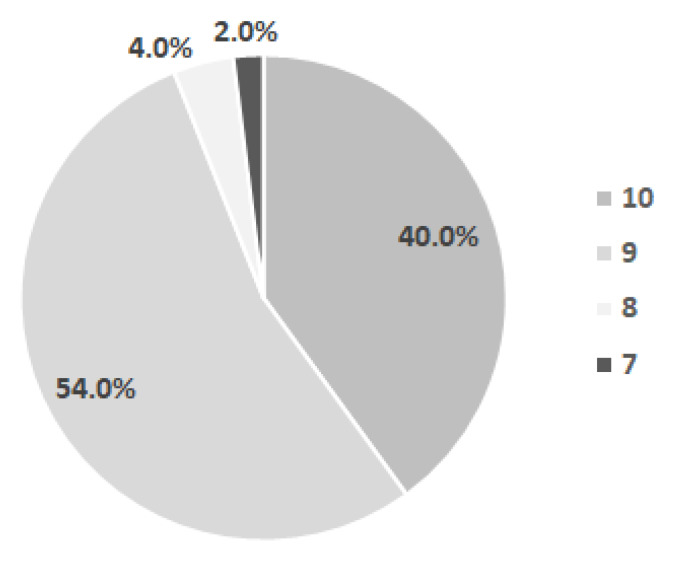
Distribution of children by the Apgar score.

Of the total number of children for whom the Apgar score was completed, more than half received the score of 9 (54%) and a good portion (40%) received the Apgar score of 10. Only three children received a lower Apgar score (7 and 8) (Figure 3).

In terms of mother’s age at birth, the highest frequency of babies born was at 29 and 30 years, and a slight decrease followed, then remaining approximately constant until the age of 34. The minimum age was 22 years and the maximum was 42 years. Only the child whose mother was 42 years old was conceived by in vitro fertilisation. The average age of mothers at birth was 30.22 years, and the standard deviation was 4.03 (Table 1).

The minimum weight recorded at birth was 2500 g and the maximum was 4200 g, while the average weight was about 3300 g. The minimum length was 45 cm, the maximum length was 56 cm, and the average length was about 51 cm (Table 1). The height and weight were recorded at one month, at two months, at three months, and at six months (the period of the maximum postnatal somatic growth), and then these data were recorded every six months until the end of the eruption of temporary teeth, which is considered to be at the age of two and a half years (Table 1).

In regard to diet, the authors can divide the sample into two groups: children who were breast-fed (74.3%) and children who did not benefit from this type of diet (11.4%).

The group of breast-fed children was subdivided into two categories: children who were breast-fed for six months (55.77%) and children who were breast-fed for less than six months (44.23%).

The diet was, in some children, exclusively natural, and, in others, it was mixed (requiring the addition of synthetic milk). The total number of months of exclusive breastfeeding was 313 months for 52 children, which is an average of 6.01 months for each child. The total number of months of mixed feeding for the 52 children was 78 months, which is an average of 1.5 months. The average number of months of breastfeeding was 7.51 months.

All the data presented above are important in order to be able to best characterise the studied group from the point of view of the general health status and general somatic growth, and to be able to determine the correlations with certain parameters that could influence dental eruption.

### 3.2. Specific Data for Primary Teeth Eruption

The data presented below are specific for the eruption of primary teeth:

1. the average age of eruption of each temporary tooth (Table 2); the teeth are ordered upwards by the age of eruption, which leads to the determination of the average sequence of deciduous teeth eruption, represented by the same table;
2. the average age for the eruption of pairs of homologous deciduous teeth, determined on the total number of dental units of the same type (cumulated from the left and right sides) (Table 3); the groups of teeth are in ascending order, depending on the age of eruption, thus resulting in the average sequence of eruption of deciduous teeth;
3. the average age for the eruption of pairs of homologous deciduous teeth, determined separately for the two sexes (Table 4);
  - The average eruption age for most deciduous teeth is slightly decreased for girls compared to boys, with the exception of the upper central incisors and lower lateral incisors. The differences between the sexes are insignificant for most of the groups of teeth compared (the value *p* = 0.757), except for the maxillary canines (*p* = 0.047) and mandibular canines (*p* = 0.018), and for the lower deciduous second molars (*p* < 0.001), which are significantly increased in boys compared to girls.

4. the comparisons between the average eruption ages of the deciduous teeth by sex were made using the unpaired *t*-test, and those between the two hemiarches, respectively between the two arches (maxilla and mandible) were made using the paired *t*-test;
  - The differences between the two hemiarches (right and left) are insignificant (*p* = 0.197), globally and also for every pair of teeth (*p* > 0.05).
  - The differences between the maxilla and the mandible are significantly (*p* = 0.019 value) increased in the maxilla. The application of the same test on groups of teeth identified the earlier eruption of the central mandibular incisors and of the second mandibular molars, as well as of the maxillary lateral incisors.

The average eruption ages of the deciduous teeth are significantly higher in the case of the maxilla for the 5.5–8.5 pair of teeth (*p* = 0.008), for the 5.1–8.1 and 6.1–7.1 pairs of teeth (*p* < 0.001), and for the 6.5–7.5 pair of teeth (*p* = 0.005); the average eruption ages of the temporary teeth is significantly lower in the case of the maxilla for the 5.2–8.2 pair of teeth (*p* = 0.017) and for the 6.2–7.2 pair of teeth (*p* = 0.001). In the rest of tooth pairs, the differences between the maxilla and mandible are insignificant.

5. the number of primary teeth present in the child’s mouth at different ages was also determined: 2.38 ± 1.39 at 6 months, 6.82 ± 3.10 at 12 months, 12.90 ± 2.74 at 18 months, and 16.85 ± 3.05 at 24 months.
6. the mean eruption age of the first primary tooth, independently of the exact type of tooth which erupted first, is 7.07 ± 1.99 months (Min 3 Months, Max 13 months).
7. correlations between the eruption timing of the first primary tooth and the weight and height at birth and at six months, the mother’s age at birth and the total number of breastfeeding months are direct, weak and insignificant, and the correlation between the number of teeth erupted at one year and the height at one year is weak, inverse and significant for the studied sample (Table 5).
8. a comparison between the timing of eruption of the first primary tooth according to the number of previous births (7.0 ± 1.70 for the first birth vs. 7.2 ± 2.49 for the second), and a comparison according to the type of diet have been made (7.22 ± 1.98 for natural diet vs. 7.20 ± 2.28 for artificial diet); the unpaired *t*-test was used and the differences were insignificant for both comparisons made (*p* = 0.665 for the first one and *p* = 0.983 for the second one).

## 4. Discussion

### 4.1. Updating the Reference Values

Taking into account the fact that the group was made up predominantly of children with good health, born in 2008–2010, without congenital diseases or problems at birth, with a predominance of the values 9 and 10 for the Apgar score, breast-fed or with mixed feeding in a proportion of 74.3% (over half of them being breast-fed for more than 6 months), with anthropometric parameters at birth, and along the way, situated within normal limits, the authors considered the average values determined as being able to characterise the current population. In order to develop the up-to-date reference values for the Romanian population, a larger data pool should be gathered in different regions around the country.

According to the results obtained, deciduous teeth eruption begins, on average, immediately after reaching the age of 7 months (7.07 months), and ends, on average, immediately after the age of 26 months (26.14 months). According to previous results from the national literature, E. Bratu determined the onset of dental eruption for the first studied group as occurring at the age of 7 months, and, for the second group studied, at the age of 9 months. The end of dental eruption was at 26 months for both samples [4]. We can assume that the age of onset and end of dental eruption has remained approximately the same in contemporary children.

Following the comparisons made with the two studies from national literature and with other studies in the international literature, as well as with the standards presented in paediatric dentistry–orthodontics treatises [4,38,39,40,41,42], the authors came to the conclusion that there are differences regarding the average eruption ages of deciduous teeth recorded in different populations and at different periods of time (Table 6 and Table 7).

**Figure 4:**
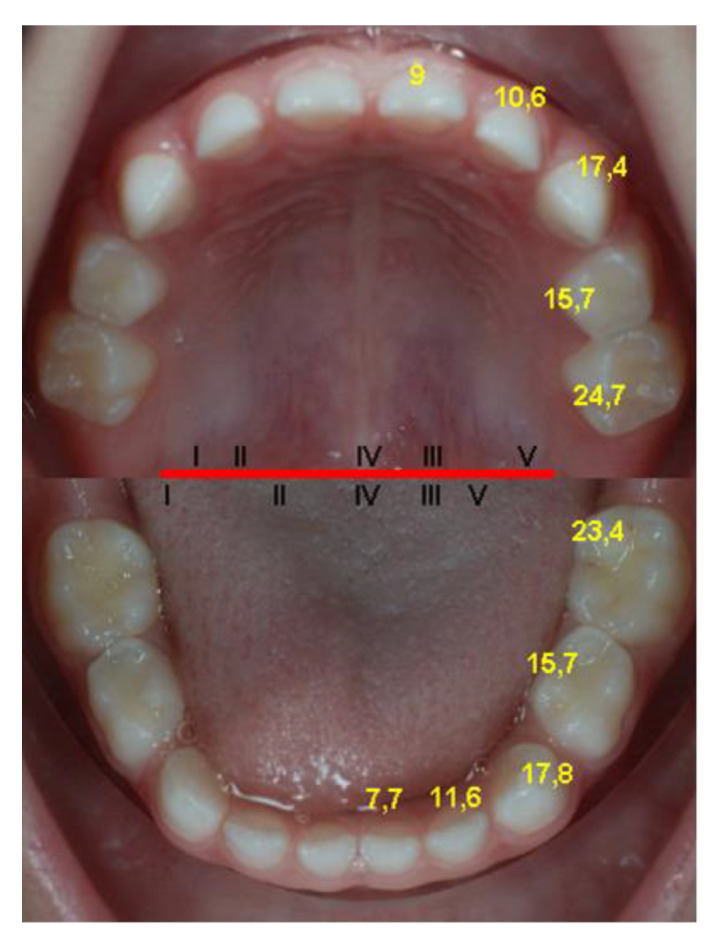
Reference values of timing (months) and sequence of primary teeth eruption (tooth numbers of the upper and lower jaw quadrant, in relationship to the red line) in our sample.

The eruption sequence is the same in all the studies, with very few exceptions, among which we mention the study conducted on the A group by E. Bratu, where the primary central maxillary incisors erupt before the mandibular ones, the rest of the sequence being preserved. The primary lateral maxillary incisors erupt before the mandibular incisors in all cases, with the exception of the standards described by Stöckli and those described by Peltomäki, where the eruption ages are not differentiated between them. Proffit, however, differentiates between them. The primary canines and first primary molars of the maxilla erupt at about the same time as the mandibular ones, and the primary mandibular secondary molars erupt before the maxillary ones (Figure 4). Determining almost the same sequence of eruption proves that this is under a strong genetic control, the environmental factors being able to influence to a greater extent the age of eruption of different groups of teeth or individual dental units [38,39,41].

The average ages of deciduous tooth eruption vary depending on the population recorded and the time when the study was conducted, but the average sequence of eruption is the same, with very few exceptions. The individual eruption ages of deciduous teeth are under individual genetic control (which also differs depending on ethnicity) and under the influence of environmental factors. The differences between them lead to the appearance of individual eruption sequences (polymorphism of the deciduous teeth eruption sequence), which fail to disturb the average eruption sequence, even when it is determined by studying a small group of 70 children.

The average age of eruption of the mandibular primary central incisors determined by this study was almost identical to that determined 30 years ago by E. Bratu, and was also close to the values described in the treatises or determined by some studies (after W. Proffit, after P. Stöckli, after T. Peltomäki, and according to the 1984 and 2003 studies in Australia, and those in 1984 in Canada and in 1994 in Spain). The values obtained from other studies, including those obtained on the study group B of E. Bratu, are increased for this tooth.

The eruption age of the primary central maxillary incisor was decreased compared to the standards described in the three treatises mentioned, and very similar to those determined in 1984 and in 2003 in Australia and in 1984 in Canada, and is among the values described by E. Bratu for the two groups included in her study.

The values of the primary maxillary and mandibular lateral incisors were very close to the values of E. Bratu in group A for these teeth, slightly lower than the values described in the treatises and in some studies.

The first primary maxillary and mandibular molars erupted approximately at the same time, and the value was similar to that described in the treatises and some studies, and decreased compared to the values of E. Bratu. Primary canines erupted approximately at the same time, the maxillary ones with the mandibular ones, but much earlier compared to the results from the treatises or to other studies. The same was found for the second primary molars, the mandibular ones erupting before the maxillary ones, similarly to the other studies (Table 6 and Table 7). The earlier eruption of the primary canines and second primary molars that was observed in the current sample leads us to propose the clarification of this result through further studies.

Following our data analysis, the authors came to the following conclusions: at the age of one year, most children have at least six deciduous erupted incisors, at the age of 18 months most children have all the incisors and the first primary maxillary and mandibular molars, at the age of 20 months children have the primary canines erupted, at the age of 24 months eruption is already completed in some children, and at 2.2 years (26 months) most children have all the deciduous teeth on the arches.

The differences between the two hemiarches in terms of age of eruption are not significant, cancelling each other out. This does not necessarily imply the simultaneous eruption on the two hemiarches of the two teeth belonging to the group, and there may be differences of up to several months. Differences greater than a month can be considered rare, and the most frequent and main differences were found in the group of primary mandibular lateral incisors.

The intermaxillary differences were significant only for the primary central incisors and second molars, the mandibular ones erupting significantly earlier than the maxillary ones. In some cases (17.46% for our group), one or both maxillary incisors may erupt before the mandibular ones. Thus, the eruption scheme presented for group A in 1979 is valid for 17.46% of the children in our group. The sequence determined on group B from 1978 is valid for the rest of the children in our group, with one modification: the first primary molars and the primary maxillary and mandibular canines erupted at about the same time for the two arches in our sample.

The maxillary and mandibular canines and second mandibular molars erupt faster in girls compared to boys. The authors thus consider that between one and a half and two years, there is an acceleration of the dental eruption in girls compared to boys. E. Bratu found earlier eruption of these teeth in girls, except for mandibular canines.

### 4.2. Study Limitation

Due to the current context regarding the lack of studies on primary teeth eruption in Romania, we decided to conduct a study using the longitudinal method of evaluation. This method has the main advantage that studies can be applied to smaller population groups and provide a lot of information regarding the timing and sequence of tooth eruption. On the other hand, the advantages of cross-sectional studies consist of an accurate data determination by the dentists, including the degree of eruption of each tooth and a faster and more accurate determination of the number of teeth at a certain age. The disadvantages of these studies are: larger samples are needed in order to be able to capture the moments of eruption of all deciduous teeth; and the eruption sequence can be determined only by taking into account the average eruption ages of all teeth, as only one eruption sequence may be identified, namely the average eruption sequence. The biggest disadvantages of longitudinal studies, using the questionnaire method completed by parents, are the errors that can occur and the risk of data loss during the study.

### 4.3. Future Perspectives

In the evaluations carried out using the method of questionnaires addressed to parents, there is a need for a better involvement on their part, and for a closer collaboration between dentists and neonatologists or paediatricians, who could best mediate the relationship between the parents and doctors involved in the research for updating dental eruption standards. More parents should be trained in how to record eruption data, and communication with them, as well as data collection, should be undertaken frequently. All children participating in the study should be examined bimonthly, monthly, or at least every three months by the researcher, in accordance to previous similar studies [11,43]

Among the future perspectives, the authors also consider the development of one study with a similar design with the possibility of interregional application.

## 5. Conclusions

According to the findings of this study, the first deciduous tooth that erupts is the lower central incisor, followed by the upper central incisor, upper lateral incisor, lower lateral incisor, first primary molars, upper canine, lower canine, lower second primary molar, and upper second primary molar. The differences between the two hemiarches (right and left) are insignificant.

There was an acceleration of the eruption in the central mandibular incisors and the second mandibular molars compared to the maxillary counterparts, and in the maxillary lateral incisors compared to the mandibular counterparts. The average eruption age for most deciduous teeth was slightly decreased in girls compared to boys, and the differences were significant only for the primary maxillary and mandibular canines and the deciduous inferior second molars. No significant difference was found between the timing of eruption of the first primary tooth according to the number of previous births and to the type of diet.

## Figures and Tables

**Table 1:** Table of characteristics from birth to 30 months.

Variable	N	Mean ± Std. Deviation	Minimum	Maximum
**The age of the mother at birth**				
No. of mothers	60	30.2 ± 4.03	22	42
Weight and height at birth				
Weight(W) (kg)	64	3321.7 ± 401.53	2500	4200
Height(H) (cm)	63	50.7 ± 2.02	45	56
**Weight (kg) at eight different times (months)**				
W1	45	4045.7 ± 527.1	2700	5100
W2	48	4965.8 ± 701.56	3200	6800
W3	45	5659.7 ± 780.44	3500	7630
W6	49	7486.0 ± 1041.99	3900	9400
W12	46	9740.0 ± 1332.88	5930	12,500
W18	26	11,498.4 ± 1998.3	6300	15,500
W24	19	12,833.6 ± 1621.89	9010	16,200
W30	6	14,800.0 ± 1649.24	13,000	17,800
**Height (cm) at eight different times (months)**				
H1	34	54.0 ± 2.39	48	58
H2	38	57.3 ± 3.02	50	64
H3	36	60.4 ± 4.29	50	72
H6	39	67.2 ± 5.2	52	86
H12	37	75.6 ± 5.69	60	92
H18	22	81.4 ± 7.06	62	94
H24	17	89.2 ± 5.43	80	100
H30	5	99.4 ± 8.35	90	110

**Table 2:** The age and sequence of eruption of each primary tooth.

Tooth	N	Age (Months)		
		Mean ± Std. Deviation	Minimum	Maximum
8.1	65	7.7 ± 2.28	3	14
7.1	66	7.7 ± 2.33	3	13
6.1	62	9.0 ± 2.36	3	17
5.1	61	9.0 ± 2.25	4	18
5.2	50	10.5 ± 2.53	6	16
6.2	56	10.8 ± 3.31	6	26
8.2	52	11.5 ± 3.84	6	28
7.2	53	11.7 ± 3.97	6	29
6.4	42	15.6 ± 2.69	11	21
8.4	39	15.7 ± 3.24	10	23
5.4	40	15.7 ± 3.01	11	23
7.4	44	15.7 ± 2.95	9	23
6.3	29	17.3 ± 2.99	10	22
5.3	29	17.4 ± 2.96	10	22
7.3	26	17.7 ± 3.19	10	24
8.3	27	18.0 ± 3.37	10	24
7.5	14	23.4 ± 2.24	20	29
8.5	14	23.5 ± 2.71	19	29
6.5	11	24.5 ± 2.12	21	29
5.5	10	25.0 ± 2.67	21	30

**Table 3:** The age and sequence of eruption of the groups of two deciduous teeth.

Teeth	N	Age (Months)		
		Mean ± Std. Deviation	Minimum	Maximum
7.1–8.1	131	7.7 ± 2.31	3	14
5.1–6.1	123	9.0 ± 2.29	3	18
5.2–6.2	106	10.6 ± 2.96	6	26
7.2–8.2	105	11.6 ± 3.87	6	29
5.4–6.4	82	15.7 ± 2.83	11	23
7.4–8.4	83	15.7 ± 3.07	9	23
5.3–6.3	58	17.4 ± 2.95	10	22
7.3–8.3	53	17.8 ± 3.25	10	24
7.5–8.5	28	23.4 ± 2.44	19	29
5.5–6.5	21	24.7 ± 2.35	21	30

**Table 4:** The average eruption ages of the deciduous teeth by sex.

Group of Teeth	Female				Male			
	N	Mean ± Std. Deviation	Minimum	Maximum	N	Mean ± Std. Deviation	Minimum	Maximum
5.1–6.1	61	9.0 ± 2.814	3	18	62	9.0 ± 1.66	5	13
5.2–6.2	55	10.6 ± 3.56	6	26	51	10.7 ± 2.16	7	15
5.3–6.3	34	16.7 ± 3.35	10	22	24	18.3 ± 1.99	15	22
5.4–6.4	47	15.3 ± 3.04	11	23	35	16.1 ± 2.51	13	22
5.5–6.5	13	24.4 ± 1.66	22	28	8	25.3 ± 3.24	21	30
7.1–8.1	67	7.5 ± 2.6	3	14	64	8.0 ± 1.97	5	13
7.2–8.2	55	12.0 ± 4.63	7	29	50	11.2 ± 2.79	6	16
7.3–8.3	31	16.9 ± 3.69	10	23	22	19.1 ± 1.99	16	24
7.4–8.4	47	15.3 ± 3.33	9	23	36	16.1 ± 2.71	12	23
7.5–8.5	20	22.5 ± 1.91	19	26	8	25.8 ± 2.12	24	29

**Table 5:** The correlation between the timing of eruption of the first primary tooth and for the number of teeth at one year.

Variable	Pearson Coefficient (r)	*p* ^significance^
*Correlation between the eruption age of the first primary tooth and…*		
Weight at birth	0.174	0.193 ^is^
Height at birth	0.157	0.239 ^is^
Weight at 6 months	0.198	0.183 ^is^
Height at 6 months	0.179	0.290 ^is^
Mother’s age	0.053	0.706 ^is^
Natural diet	0.19	0.190 ^is^
*Correlation between the number of teeth erupted at 1 year and…*		
Weight at 12 months	−0.048	0.768 ^is^
Height at 12 months	−0.394	0.023 ^s^

Legend: ^is^—insignificant correlation; ^s^—significant correlation.

**Table 6:** Comparison of the results obtained with standard values, presented in the pedodontics-orthodontics treatises and results of other studies [38,39,40,41,42].

Teeth	Mean Age (Months) of Primary Teeth Eruption						
	Current Study	Peltomäki 2009 [38]	Proffit 2007 [39]	Stöckli 2001 [41]	Australia 2003 [42]	Australia 2010 [42]	India 2009 [40]
7.1–8.1	7.73	7	8	7	7.2	8.6	10.72
5.1–6.1	8.98	10	10	10	9	10.8	12.03
5.2–6.2	10.62	12	11	12	10.4	12.2	13.46
7.2–8.2	11.59	12	13	12	12.8	14.1	12.61
5.4–6.4	15.66	16	16	16	15.3	15.9	17.26
7.4–8.4	15.68	16	16	16	16	16.6	19.02
5.3–6.3	17.38	20	19	20	18.2	19.3	21.18
7.3–8.3	17.81	20	20	20	18.6	19.8	22.10
7.5–8.5	23.43	28	27	30	26.1	26.9	27.18
5.5–6.5	24.71	28	29	30	26.7	27.8	28.68

**Table 7:** Comparison of the average values obtained with average values from other studies [4,42].

Teeth	Mean Age of Primary Teeth Eruption						
	Current Study	Bratu Sample A 1979 [4]	Bratu Sample B 1978 [4]	Japan 1970 [42]	Australia1984 [42]	Canada 1984 [42]	Spain 1994 [42]
7.1–8.1	7.73	7.7	8.65	9.38	7.1	7.18	7.20
5.1–6.1	8.98	7	9.56	10.89	8.9	9.03	9.42
5.2–6.2	10.62	10.9	11.82	12.70	10.2	10.19	10.66
7.2–8.2	11.59	11.6	13.23	13.87	11.8	12.13	12.26
5.4–6.4	15.66	15	16.95	17.30	15	15.13	15.28
7.4–8.4	15.68	16.5	17.45	17.91	15.2	15.01	15.70
5.3–6.3	17.38	20.4	20.9	18.11	18.3	18.04	18.70
7.3–8.3	17.81	21.9	21.12	19.74	18.8	18.34	19.03
7.5–8.5	23.43	24.6	24.75	27.19	26	26.40	25.47
5.5–6.5	24.71	25.7	25.75	28.63	26.6	27.48	26.77

## Data Availability

The data presented in this study are available on request from the corresponding author.
